# Memory B cell development elicited by mRNA booster vaccinations in the elderly

**DOI:** 10.1084/jem.20230668

**Published:** 2023-06-27

**Authors:** Zijun Wang, Frauke Muecksch, Raphael Raspe, Frederik Johannsen, Martina Turroja, Marie Canis, Mohamed A. ElTanbouly, Gabriela S. Silva Santos, Brianna Johnson, Viren A. Baharani, Rachel Patejak, Kai-Hui Yao, Bennett J. Chirco, Katrina G. Millard, Irina Shimeliovich, Anna Gazumyan, Thiago Y. Oliveira, Paul D. Bieniasz, Theodora Hatziioannou, Marina Caskey, Michel C. Nussenzweig

**Affiliations:** 1 Laboratory of Molecular Immunology, The Rockefeller University, New York, NY, USA; 2 Laboratory of Retrovirology, The Rockefeller University, New York, NY, USA; 3 Department of Infectious Diseases, Virology, University of Heidelberg, Heidelberg, Germany; 4 Howard Hughes Medical Institute, Maryland, MD, USA

## Abstract

Despite mRNA vaccination, elderly individuals remain especially vulnerable to severe consequences of SARS-CoV-2 infection. Here, we compare the memory B cell responses in a cohort of elderly and younger individuals who received mRNA booster vaccinations. Plasma neutralizing potency and breadth were similar between the two groups. By contrast, the absolute number of SARS-CoV-2–specific memory B cells was lower in the elderly. Antibody sequencing revealed that the SARS-CoV-2–specific elderly memory compartments were more clonal and less diverse. Notably, memory antibodies from the elderly preferentially targeted the ACE2-binding site on the RBD, while those from younger individuals targeted less accessible but more conserved epitopes. Nevertheless, individual memory antibodies elicited by booster vaccines in the elderly and younger individuals showed similar levels of neutralizing activity and breadth against SARS-CoV-2 variants. Thus, the relatively diminished protective effects of vaccination against serious disease in the elderly are associated with a smaller number of antigen-specific memory B cells that express altered antibody repertoires.

## Teaser

Wang et al. examined antibody and memory B cell responses to SARS-CoV-2 mRNA booster vaccination in the elderly. Their analysis revealed a small but significant decrease in memory B cell numbers with an altered antibody repertoire that may contribute to a higher risk for severe disease in the elderly.

## Introduction

The COVID-19 pandemic disproportionately affected the elderly population, which was among the most prone to hospitalization and death (de Lusignan et al., 2020; Hewitt et al., 2020; Mueller et al., 2020; Williamson et al., 2020). Fortunately, mRNA vaccination resulted in a substantial decline in COVID-19–related hospitalizations and deaths in the elderly (Britton et al., 2022; Havers et al., 2022b; Moghadas et al., 2021). However, the most pronounced increase in hospitalization and mortality associated with emerging variants in vaccinated individuals was among the elderly (Havers et al., 2022a). Cohorts of younger individuals that receive a third vaccine dose are believed to be protected from hospitalization after infection with SARS-CoV-2 variants in part because the booster dose increases diversity and the number of neutralizing antibody-producing memory B cells that can rapidly be recalled upon challenge (Andrews et al., 2022; Barda et al., 2021; Lustig et al., 2022; Muecksch et al., 2022; Tang et al., 2022; Tartof et al., 2022; Thompson et al., 2022). However, little is known about the memory B cell response in the elderly.

Here, we examined the memory B cell responses in a cohort of elderly individuals whose median age was 77 yr and who received three or four doses of an mRNA vaccine. Elderly vaccinees develop a smaller number of memory B cells that are less diverse and more clonal than younger vaccinees. In addition, the relative distribution of the epitopes targeted by memory antibodies in the elderly differs. Despite these differences, the individual potency of the memory antibodies is comparable in the two age groups.

## Results

**Figure S1.**
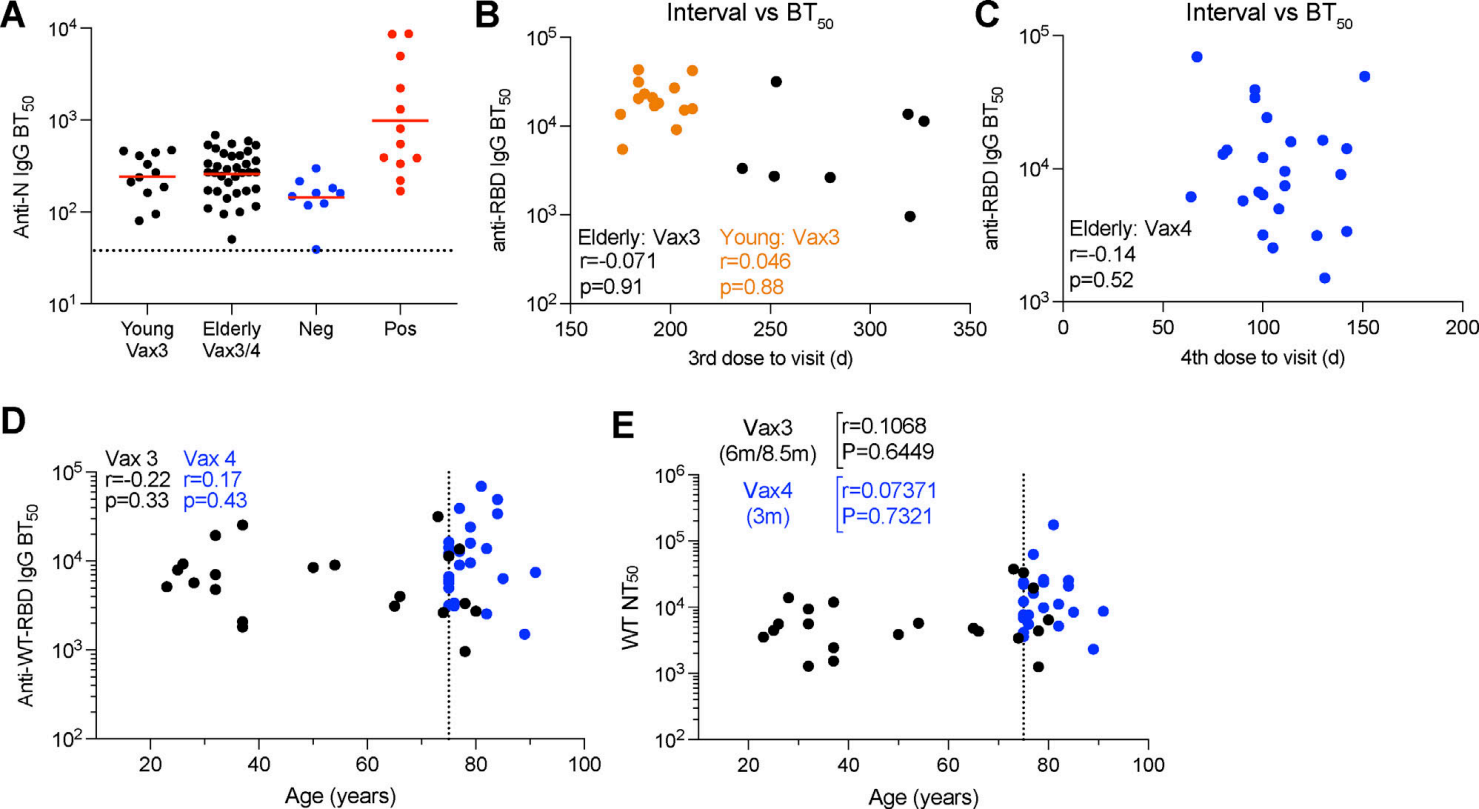
**Plasma ELISA. (A)** BT_50_s for anti-nucleocapsid (N) IgG. **(B and C)** BT_50_s for anti-RBD IgG plotted against intervals between vaccine doses and blood collection. **(D and E)** Age (x axis) plotted against BT_50_s for anti-RBD IgG (y axis; D) or NT_50_s for WT (y axis; E). All experiments were performed at least in duplicate and repeated twice. The elderly Vax4 value is shown in blue. Statistical significance was determined by two-tailed Spearman’s correlation.

**Figure 1.**
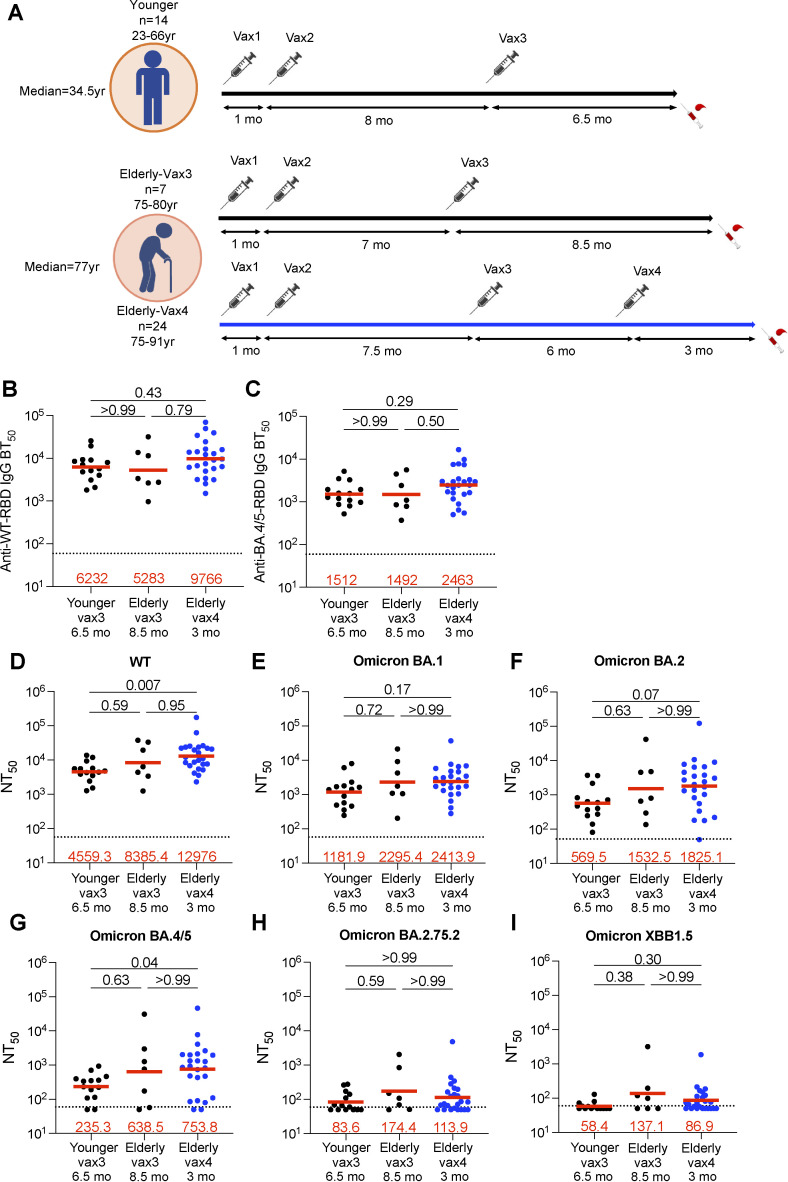
**Plasma ELISAs and neutralizing activity. (A)** The diagram shows blood donation schedules for the younger participants 6.5 mo after the third dose (top, *n* = 14), and for the elderly participants 8.5 mo after the third dose (Vax3, bottom, *n* = 7) and 3 mo after the fourth dose (Vax4, bottom, *n* = 24). **(B and C)** Graph shows half-maximal binding titer (BT_50_) for plasma IgG antibody binding to WT SARS-CoV-2 (WT) RBD (B), and Omicron BA.4/5 RBD (C). **(D–I)** Plasma neutralizing activity against indicated SARS-CoV-2 variants: (D) Wuhan-hu-1 (WT), (E) Omicron BA.1, (F) Omicron BA.2, (G) Omicron BA.4/5, (H) Omicron BA.2.75.2, and (I) Omicron XBB.1.5. The deletions/substitutions corresponding to viral variants used in D–I were incorporated into a spike protein that also includes the R683G substitution, which disrupts the furin cleavage site and increases particle infectivity. Neutralizing activity against mutant pseudoviruses was compared to a WT SARS-CoV-2 spike sequence (NC_045512), carrying R683G. All experiments were performed at least in duplicate. The elderly Vax4 datapoints are shown in blue. Red bars and values in B–I represent geometric mean values. Statistical significance in B–I was determined by two-tailed Kruskal–Wallis test with subsequent Dunn’s multiple comparisons.

Between March 24, 2022, and September 7, 2022, we enrolled a cohort of 45 individuals, divided into two groups: (1) elderly individuals (*n* = 31) who were vaccinated with three or four doses of a WT mRNA vaccine, 50% of whom were female; (2) younger individuals (*n* = 14) who received three mRNA vaccine doses (WT), 68% of whom were female (Table S1). None of the participants had a history of SARS-CoV-2 infection (Fig. S1 A) and none experienced serious adverse events after vaccination. The vaccination and blood collection schedule for the two groups is depicted in Fig. 1 A. For detailed demographic information, see Materials and methods and Table S1.

### Plasma antibody binding and neutralization

Plasma IgG responses to the SARS-CoV-2 Wuhan-Hu-1 (WT) and Omicron BA.4/5 receptor-binding domain (RBD) were measured by ELISA (Muecksch et al., 2022) 6.5–9.5 mo after a third dose irrespective of whether they received a fourth vaccine dose. There was no significant difference in IgG binding titers to WT or Omicron BA.4/5 between the elderly and younger individuals whether they received three or four vaccine doses (Fig. 1, B and C; Fig. S1, B–D; and Table S1).

Plasma-neutralizing activity was measured using HIV-1 pseudotyped with the WT SARS-CoV-2 spike protein (Cho et al., 2021; Wang et al., 2021c). The geometric mean half-maximal neutralizing titer (NT_50_) for elderly individuals after the third dose was equivalent to that of the younger individuals (Fig. 1 D and Table S1). Although it did not reach statistical significance, there was a small increase in the geometric mean NT_50_ against WT in the elderly after the fourth vaccine dose and a significant 2.8-fold increase compared with younger individuals who received a third dose (Bar-On et al., 2022; Regev-Yochay et al., 2022; P = 0.007; Fig. 1 D). Finally, there was no correlation between age and plasma-neutralizing titers (Fig. S1 D).

Plasma-neutralizing activity was also assessed against Omicron BA.1, BA.2, BA.4/5, and BA.2.75.2, and XBB.1.5 variants. The geometric mean plasma NT_50_s against the variants were not different between the elderly and younger individuals who received a third mRNA vaccine dose (Fig. 1, E–I). However, the elderly who received a fourth dose showed a 3.2-fold increase in Omicron BA.4/5 neutralizing titers when compared with younger vaccinees after three vaccine doses (Fig. 1 G). Notably, Omicron XBB.1.5 showed the highest neutralization resistance of all variants tested. We conclude that, despite advanced age, the plasma from elderly individuals who had at least three mRNA vaccine doses showed comparable neutralizing activity to plasma from younger individuals against all variants tested.

### Memory B cells

**Figure S2.**
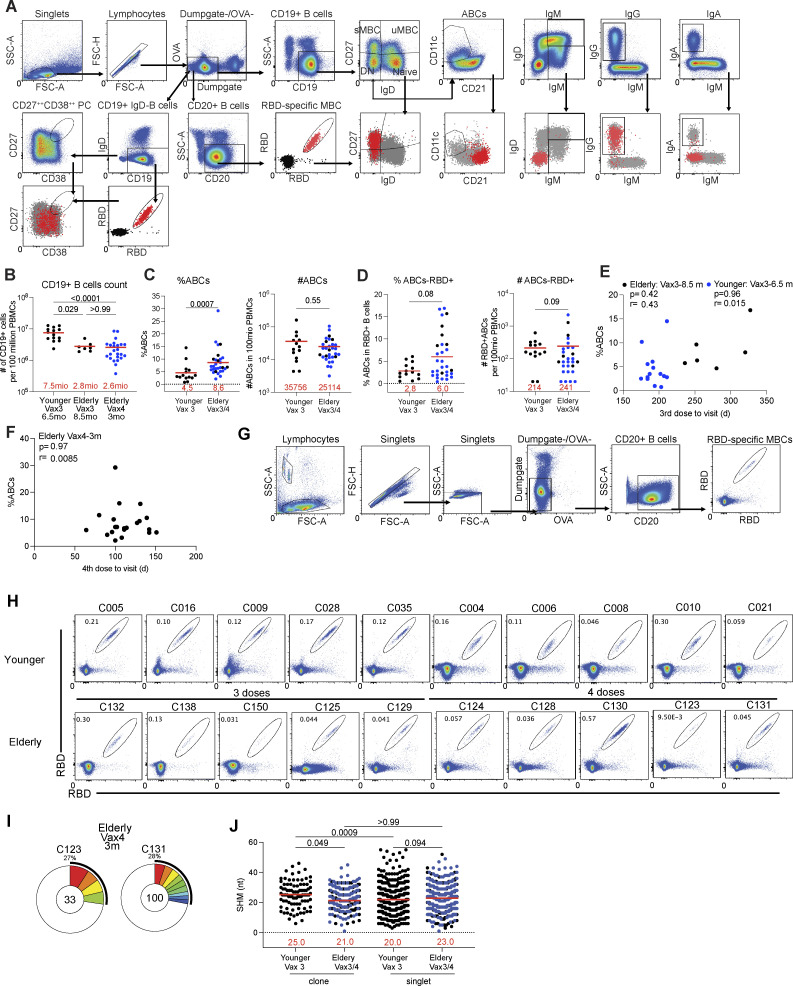
**Flow cytometry. (A)** Gating strategy for phenotyping. Gating was on lymphocytes singlets that were CD19^+^ or CD20^+^ and CD3^−^CD8^−^CD16^−^Ova^−^. Anti-IgG, IgM, IgA, IgD, CD21, CD11c, CD38, and CD27 antibodies were used for B cell phenotype analysis. Antigen-specific cells were detected based on binding to Wuhan-Hu-1 RBD-PE^+^ and RBD-AF647^+^. Gating was on lymphocyte singlets that were CD20^+^ and CD3^−^CD8^−^CD16^−^Ova^−^. Anti-IgG, IgM, and IgA antibodies were used for B cell phenotype analysis. Antigen-specific cells were detected based on binding to WT RBD-PE^+^ and RBD-AF647^+^. **(B)** Graphs show the absolute number of CD19^+^ B cells. **(C)** Graphs show the frequency and absolute number of age-associated B cells (ABCs). **(D)** Graphs show the frequency and absolute number of ABCs in WT RBD^+^ MBCs. **(E and F)** Percentage of ABCs (y axis) was plotted against intervals between vaccine doses and blood collection (x axis). **(G)** Gating strategy for single-cell sorting for CD20^+^ B cells for WT RBD-PE and RBD-AF647. **(H)** Representative flow cytometry plots indicating PE-WT-RBD and AlexaFluor-647-WT-RBD binding memory B cells from 10 younger individuals after Vax3, five elderly individuals after Vax3, and five elderly individuals after Vax4. **(I)** Pie charts show the distribution of IgG antibody sequences obtained two elderly individuals after Vax4 (in addition to the three vaccinees shown in Fig. 2 D). **(J)** Number of nucleotide somatic hypermutations (SHM) in IGHV + IGLV in WT-RBD-specific sequences from elderly or younger vaccinees. Antibodies sequences from elderly Vax4 are shown in blue. All experiments were performed at least in duplicate and repeated twice. Statistical significance in B and J was determined by two-tailed Kruskal–Wallis test with subsequent Dunn’s multiple comparisons, in C and D by two-tailed Mann–Whitney test, and in E and F by two-tailed Spearman’s correlation.

**Figure 2.**
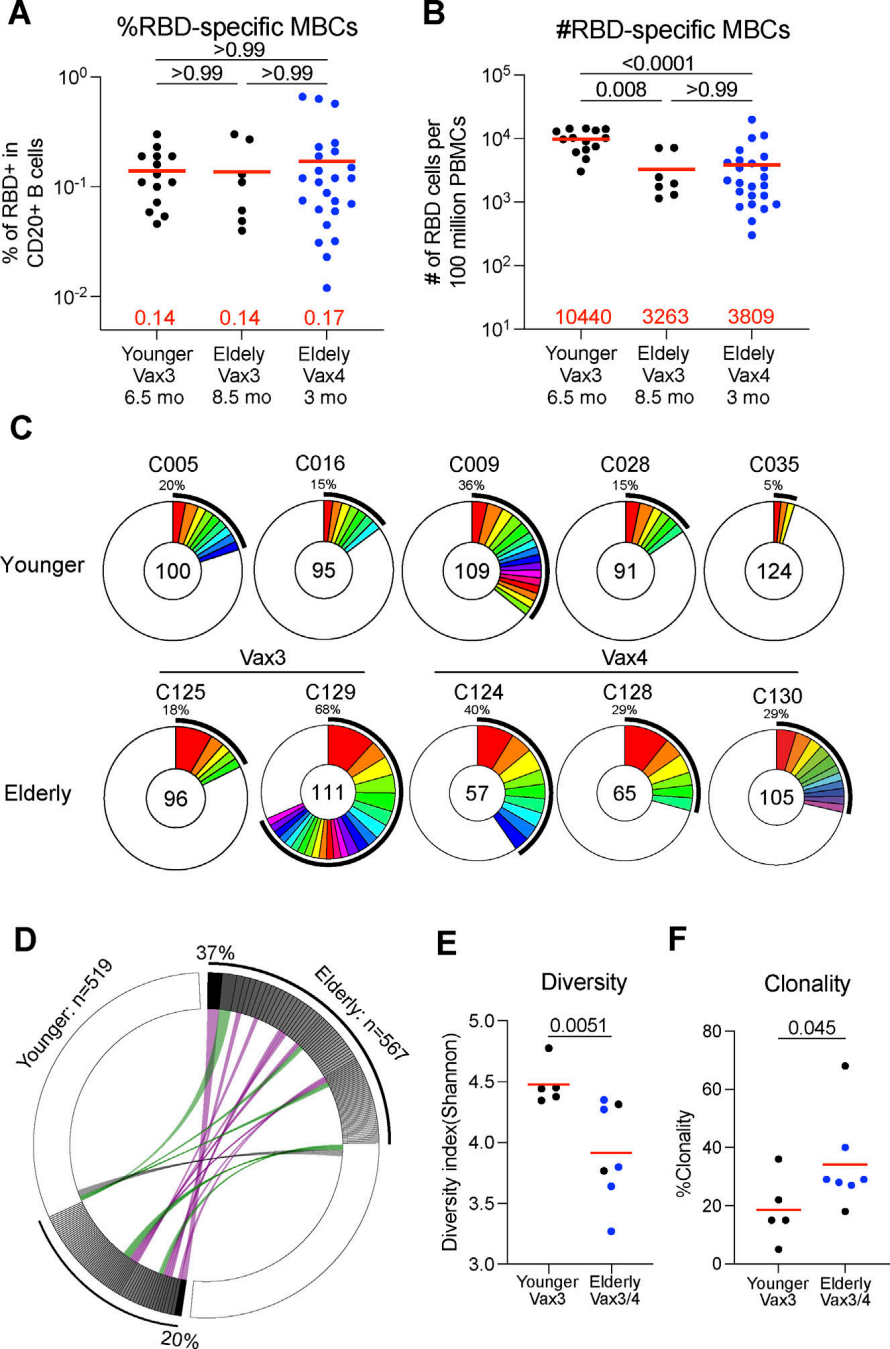
**Anti-SARS-CoV-2 RBD memory B cells after breakthrough infection. (A and B)** The frequency (A) and the number (B) of WT RBD-specific B cells are indicated for young participants after Vax3 (*n* = 24) and elderly participants 8.5 mo after Vax3 (*n* = 7) or 3 mo after Vax4 (*n* = 24, in blue). **(C)** Pie charts show the distribution of IgG antibody sequences obtained from WT-specific memory B cells from five younger individuals assayed after the third mRNA dose (Vax3); two elderly individuals after Vax3, and three elderly individuals after Vax4 (see also Fig. S3 C). The number inside the circle indicates the number of sequences analyzed for the individual denoted above the circle. Pie slice size is proportional to the number of clonally related sequences. The black outline and associated numbers indicate the percentage of clonal sequences detected at each time point. Colored slices indicate persisting clones (same IGHV and IGLV genes, with highly similar CDR3s) found at more than one time point within the same individual. Gray slices indicate clones unique to the time point. White slices indicate sequences isolated only once per time point. **(D)** Circus plot depicts the relationship between antibodies that share V and J gene segment sequences at both IGH and IGL. Purple, green, and gray lines connect related clones, clones and singles, and singles to each other, respectively. **(E and F)** The Shannon-Weiner index for diversity analysis (E) and clonality analysis (F) of the sequences from C. All experiments were performed at least in duplicate and repeated twice. The elderly Vax4 value is shown in blue. Red bars and numbers in A, B, E, and F represent mean. Statistics in A and B were determined by two-tailed Kruskal–Wallis test with subsequent Dunn’s multiple-comparisons test and in E and F by two-tailed Mann–Whitney test.

The memory B cell compartment in younger mRNA-vaccinated individuals contains a diverse collection of B cells which when challenged can produce antibodies that neutralize a variety of different viral variants (Goel et al., 2021; Goel et al., 2022; Kim et al., 2022; Muecksch et al., 2022; Sette and Crotty, 2022; Turner et al., 2021; Wang et al., 2022a; Wang et al., 2021b). To examine the memory B cell compartment in the elderly, we initially performed flow cytometry experiments using PE- and Alexa-Fluor-647 (AF647)–labeled WT RBDs (Fig. S2 A). Elderly and younger individuals showed similar relative percentages of RBD-specific memory B cells (MBCs; Fig. 2 A). However, the elderly had a smaller absolute number of B cells (P = 0.029; Fig. S2 B) and a higher relative proportion of atypical or age-associated B cells (ABCs; Cancro, 2020; P = 0.0007; Fig. S2, C and D). There was no correlation between the sampling interval and the frequency of ABCs (Fig. S2, E and F). Thus, the absolute number of circulating RBD-specific memory B cells found in the elderly was significantly lower than in the younger cohort (P = 0.008, Fig. 2 B).

**Figure S3.**
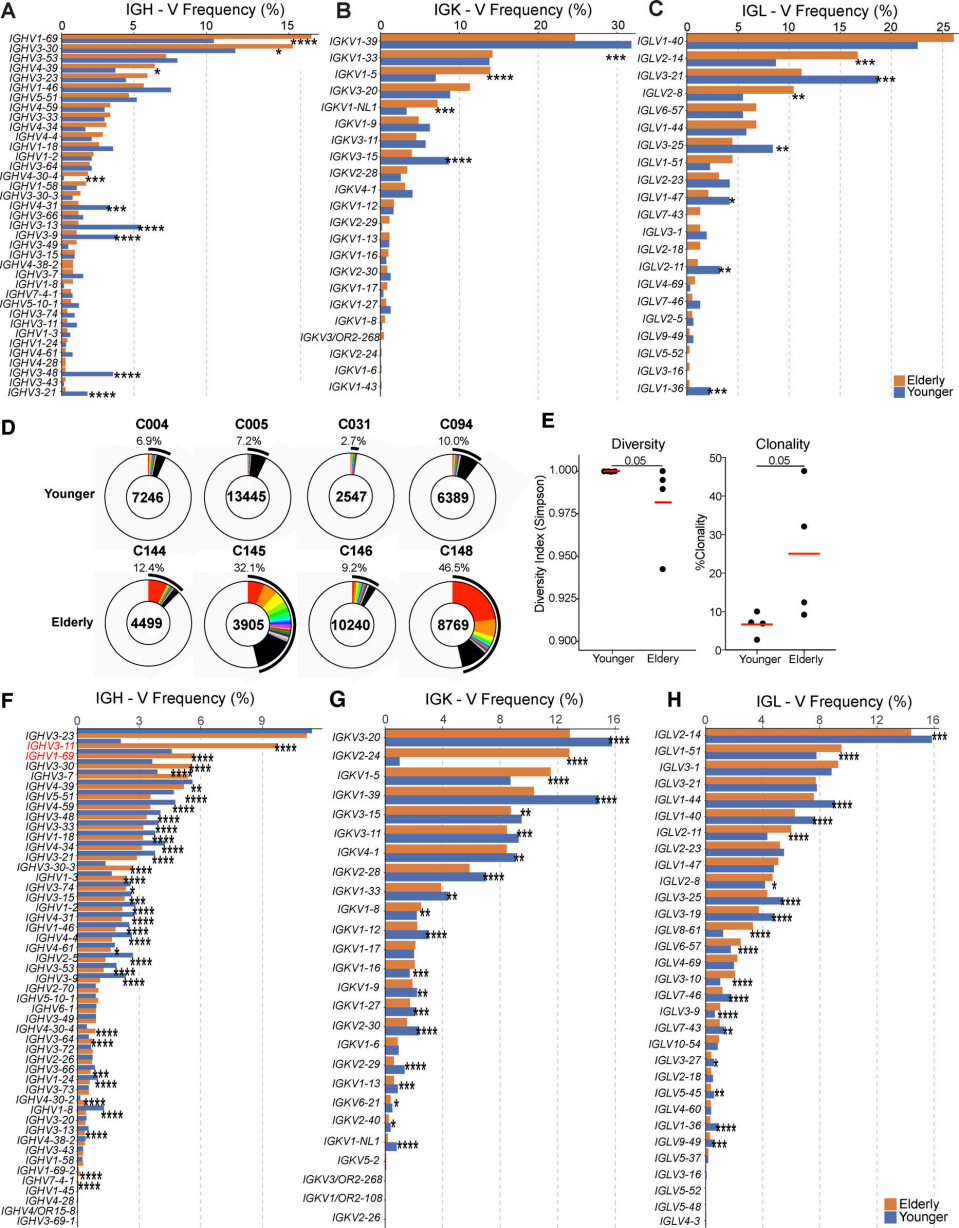
**Analysis of BCR repertoire. (A–C)** Comparison of the frequency distribution of human V genes for heavy chain and light chains of anti-RBD antibodies. The graph shows the relative abundance of human *IGHV* (A), *IGKV* (B), and *IGLV* (C) genes in antibodies obtained from elderly (orange), and younger vaccinees (blue). Statistical significance was determined by two-sided binomial test. *, P ≤ 0.05; **, P ≤ 0.01; ***, P ≤ 0.001; ****, P ≤ 0.0001. **(D–H)** Distribution of BCR repertoire and frequency distribution of V genes in circulating B cells. **(D)** Pie charts show the relative size of BCR clones as slices. The areas indicated in white represent unique BCR sequences. The number above the pie chart is the donor ID for each individual. The number in the center of the pie chart represents the number of cells assayed for each individual. **(E)** The Shannon–Weiner index for diversity analysis (left panel) and clonality analysis (right panel) of the sequences from D. **(F–H)** Graph shows the relative abundance of human *IGHV* (F), *IGKV* (G), and *IGLV* (H) genes in antibodies genes obtained from elderly (orange), and younger vaccinees (blue). Statistical significance was determined by Mann-Whitney test for E and two-sided binomial test for A–C and F–H. *, P ≤ 0.05; **, P ≤ 0.01; ***, P ≤ 0.001; ****, P ≤ 0.0001.

To compare the antibodies produced by memory B cells in the two cohorts, we obtained 567 and 519 paired heavy and light chain antibody sequences from seven elderly and five younger individuals, respectively (Fig. 2 C; Fig. S2, G–I; and Table S2). Individuals in both groups showed expanded clones of memory B cells that expressed closely related IGHV and IGLV genes (Fig. 2, C and D). However, the anti-RBD memory repertoire was less diverse in the elderly in part due to a relative increase in the number of clonally related sequences (P = 0.030, Fig. 2 E; P = 0.045, Fig. 2 F). VH1-69, VH3-30, VH4-39, and VH4-30 were over-represented, and VH4-31, VH3-13, and VH3-9 under-represented among the elderly vaccinees (Fig. S3, A–C). The biased B cell receptor (BCR) repertoire in the elderly was associated with more restricted overall V gene family member usage and increased clonality among randomly collected circulating B cells in elderly individuals (Fig. S3, D–H). Thus, the RBD-specific memory B cell antibody repertoire in elderly vaccinees is smaller and less diverse than that found in younger individuals.

**Figure 3.**
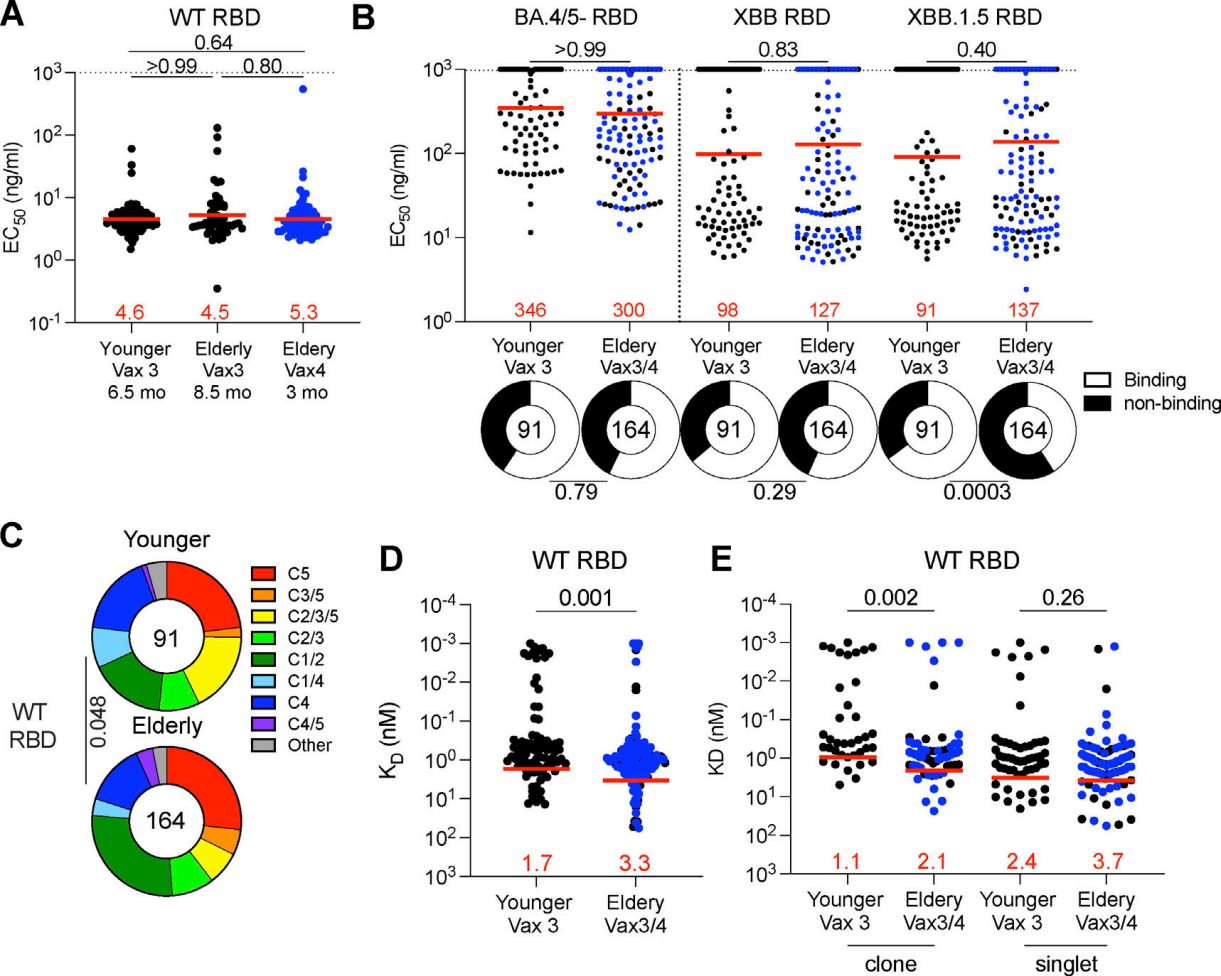
**Anti-SARS-CoV-2 RBD mAbs. (A and B)** Graphs show EC_50_ of *n* = 255 mAbs measured by ELISA against WT (A), Omicron BA.4/5, and Omicron XBB and Omicron XBB.1.5 RBD protein (B). Antibodies were obtained from memory B cells from young participants after Vax3 (Young Vax3), and elderly participants after Vax3 (Elderly Vax3) and Vax4 (Elderly Vax4). **(C)** Results of epitope mapping performed by competition BLI, comparing mAbs cloned from vaccinated younger and elderly individuals. **(D)** Graph showing *K*_D_s for WT RBD measured by BLI for antibodies cloned from young and elderly individuals. **(E)** Same as C, but for clones and singlets, separately. All experiments were performed at least in duplicate and repeated twice. Each dot represents one antibody. Antibody sequences from elderly Vax4 are shown in blue. Red bars and numbers in A and B represent the geometric mean, and in D and E represent the mean. Statistics in A (dot plot), B, and E were determined by two-tailed Kruskal–Wallis test with subsequent Dunn’s multiple-comparisons test and in D by two-tailed Mann–Whitney test, and two-sided Fisher’s exact test with subsequent Bonferroni-Dunn correction for A (ring plots).

**Figure S4.**
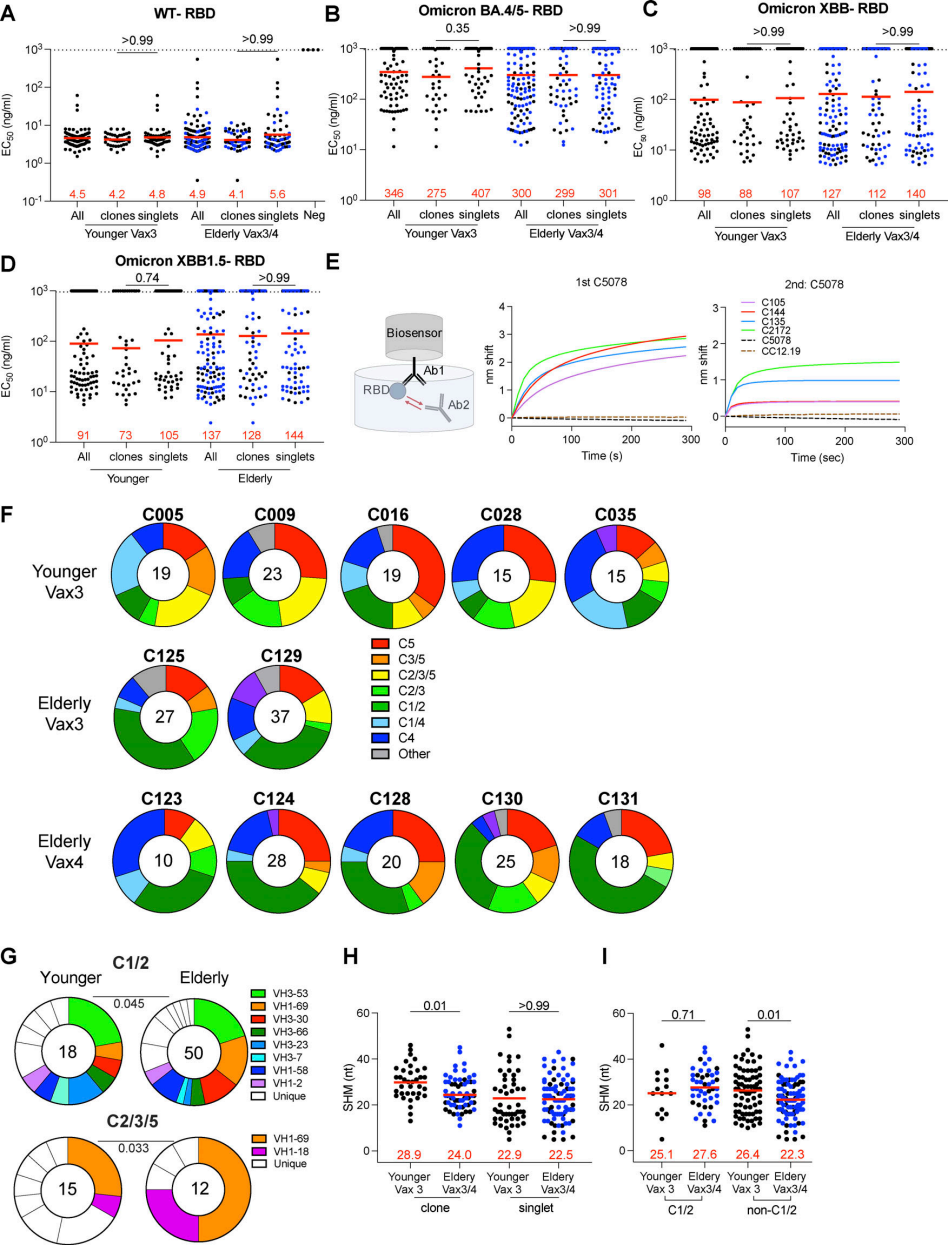
**mAb ELISAs and epitopes. (A–D)** Graphs show anti-SARS-CoV-2 binding activity EC_50_ values for all antibodies, clones, and singlets of *n* = 255 mAbs against WT-RBD (A), Omicron BA.4/5-RBD (B), Omicron XBB-RBD (C), and Omicron XBB.1.5-RBD (D). **(E)** Schematic representation of the BLI experiment for antibodies isolated from the elderly or younger vaccinees. Graphs represent the binding of the second antibody (2nd Ab) to the preformed first antibody (1st Ab)–RBD complexes. The dotted line denotes when the 1st Ab and 2nd Ab are the same. **(F)** Results of epitope mapping performed by competition BLI, comparing mAbs cloned from each participant, related to Fig. 3 C. **(G)** Results of epitope mapping performed by competition BLI, comparing mAbs belonging to Class 1/2 or Class 2/3/5, cloned from vaccinated younger individuals compared with elderly individuals. Each dot represents one antibody. Antibodies isolated from elderly Vax4 values are shown in blue. **(H)** Number of nucleotide somatic hypermutations (SHM) in IGHV + IGLV in WT-RBD–specific sequences from clones and singlets. **(I)** Number of nucleotide somatic hypermutations (SHM) in IGHV + IGLV in WT-RBD–specific sequences from Class 1/2 and non-Class 1/2 antibodies. Antibodies or antibody sequences from the elderly Vax4 are shown in blue. Red horizontal bars and numbers indicate geometric mean values. Statistical significance was determined by two-tailed Kruskal–Wallis test with subsequent Dunn’s multiple comparisons (A–D, H, and I) or by two-tailed Chi-square test (G). All experiments were performed at least twice.

To examine the specificity of the MBC antibodies, we cloned and expressed 255 mAbs. We selected one representative mAb from each clone of expanded memory B cells and at least 15 mAbs from individual memory B cells, detected only once in each participant (Table S3). 91 and 164 mAbs were obtained from younger and elderly vaccinees, respectively (Table S3). Each of the antibodies was tested for binding to WT-, Omicron BA.4/5-, XBB-, or XBB.1.5-RBDs by ELISA. All antibodies were bound to WT RBD, and there was no significant difference in the ELISA half-maximal concentrations (EC_50_s) among the groups (Fig. 3 A and Fig. S4 A). The fraction of antibodies that bound to XBB.1.5 RBD was significantly smaller in elderly than younger vaccinees (Fig. 3 B; P = 0.0003), while there was no difference in the fraction of Omicron BA.4/5 or Omicron XBB-RBD binders between the two groups (Fig. 3 B and Fig. S4, B–D). Given the similarities in binding activity between the antibodies from third and fourth dose elderly vaccine recipients, the two groups were pooled for subsequent analyses.

Memory B cell antibodies elicited after the first or second vaccine dose typically target the angiotensin-converting enzyme 2 (ACE2) binding surface of the RBD (Class 1 and 2; Barnes et al., 2020; Brouwer et al., 2020; Cho et al., 2021; Dugan et al., 2021; Lan et al., 2020; Robbiani et al., 2020; Wang et al., 2021c). By contrast, antibodies recovered from memory B cells after the third vaccine dose are more likely to target less accessible and more conserved regions of the RBD (non-Class 1 and 2; Muecksch et al., 2022). To analyze which epitopes are targeted by the memory antibodies isolated from elderly vaccinees, we performed Biolayer Interferometry (BLI) competition experiments with five antibodies that bind to different epitopes on the RBD (C105, C144, C135, C2172, and C5078 for Class 1, 2, 3, 4, and 5, respectively; Barnes et al., 2020; He et al., 2022; Muecksch et al., 2022; Rogers et al., 2020; Fig. S4 E). There was a modest but significant difference in the epitopes targeted by the memory antibodies obtained from the two groups, which was accounted for by an increased representation of Class 1/2 and decreased representation of Class 2/3/5 antibodies in the elderly (P = 0.048; Fig. 3 C and Fig. S4 F). The difference is consistent with a relative increase representation of VH1-69 and VH3-30 among RBD-binding antibodies in the elderly (Class 1/2: P = 0.045; Class 2/3/5: P = 0.033; Fig. S4 G).

The antibodies obtained from the elderly individuals showed only a slight reduction in affinity for WT RBD than the antibodies obtained from the younger individuals (affinity measurement [*K*_D_]: 1.7 vs. 3.3 nM; P = 0.001; Fig. 3 D). The decrease in affinity was mainly associated with antibodies obtained from clonally expanded B cells (P = 0.002; Fig. 3 E), which also accumulated fewer somatic hypermutations in the elderly (P = 0.01; Fig. S4 H). While the somatic mutations in Class 1/2 antibodies were similar between the two cohorts, non-Class 1/2 antibodies from the elderly carried fewer somatic mutations than the young group. Thus, our results suggest that the elderly have less adaptability in their memory antibody responses (P = 0.01; Fig. S4 I).

### Neutralization potency and breadth

**Figure 4.**
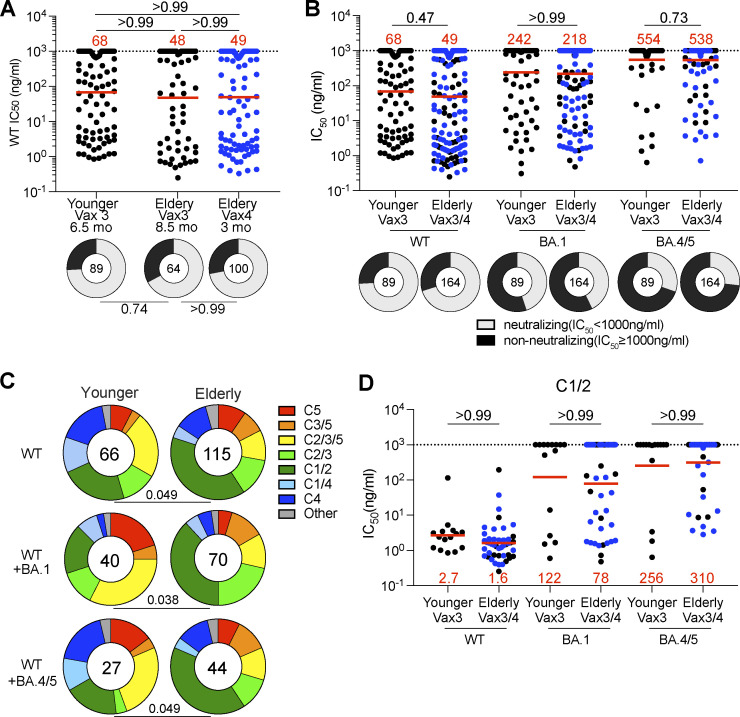
**Epitopes and neutralizing breadth. (A and B)** Graphs show anti-SARS-CoV-2 neutralizing activity of mAbs, IC_50_s value against WT, Omicron BA.1, and Omicron BA.4/5 SARS-CoV-2 pseudoviruses for all antibodies (*n* = 253). Ring plots show the fraction of neutralizing (IC_50_ < 1,000 ng/ml, gray) and non-neutralizing (IC_50_ > 1,000 ng/ml, black) antibodies against WT, Omicron BA.1, and Omicron BA.4/5 SARS-CoV-2 pseudoviruses. Number in inner circles indicates number of antibodies tested. **(C)** Results of epitope mapping performed by competition BLI, comparing mAbs cloned from vaccinated younger and elderly individuals. Pie charts show the distribution of the antibody classes among WT-neutralizing antibodies (upper panel), WT + Omicron BA.1-neutralizing antibodies (middle panel), or WT + Omicron BA.4/5-neutralizing antibodies (lower panel). **(D)** Graphs showing IC_50_ neutralization activity of Class 1/2 antibodies among all antibodies in A. The deletions/substitutions corresponding to viral variants used in A, B, and D were incorporated into a spike protein that also includes the R683G substitution, which disrupts the furin cleavage site and increases particle infectivity. Neutralizing activity against mutant pseudoviruses was compared to a WT SARS-CoV-2 spike sequence (NC_045512), carrying R683G. All experiments were performed at least in duplicate and repeated twice. Each dot represents one antibody. Antibody sequences from elderly Vax4 are shown in blue. Red bars and values in A, B, and D, represent geometric mean values. Statistics were determined by two-tailed Kruskal–Wallis test with subsequent Dunn’s multiple comparisons (A, dot plot), two-sided Fisher’s exact test with subsequent Bonferroni–Dunn correction (A, ring plots), Mann–Whitney test (B and D), and two-tailed Chi-square test (C).

**Figure S5.**
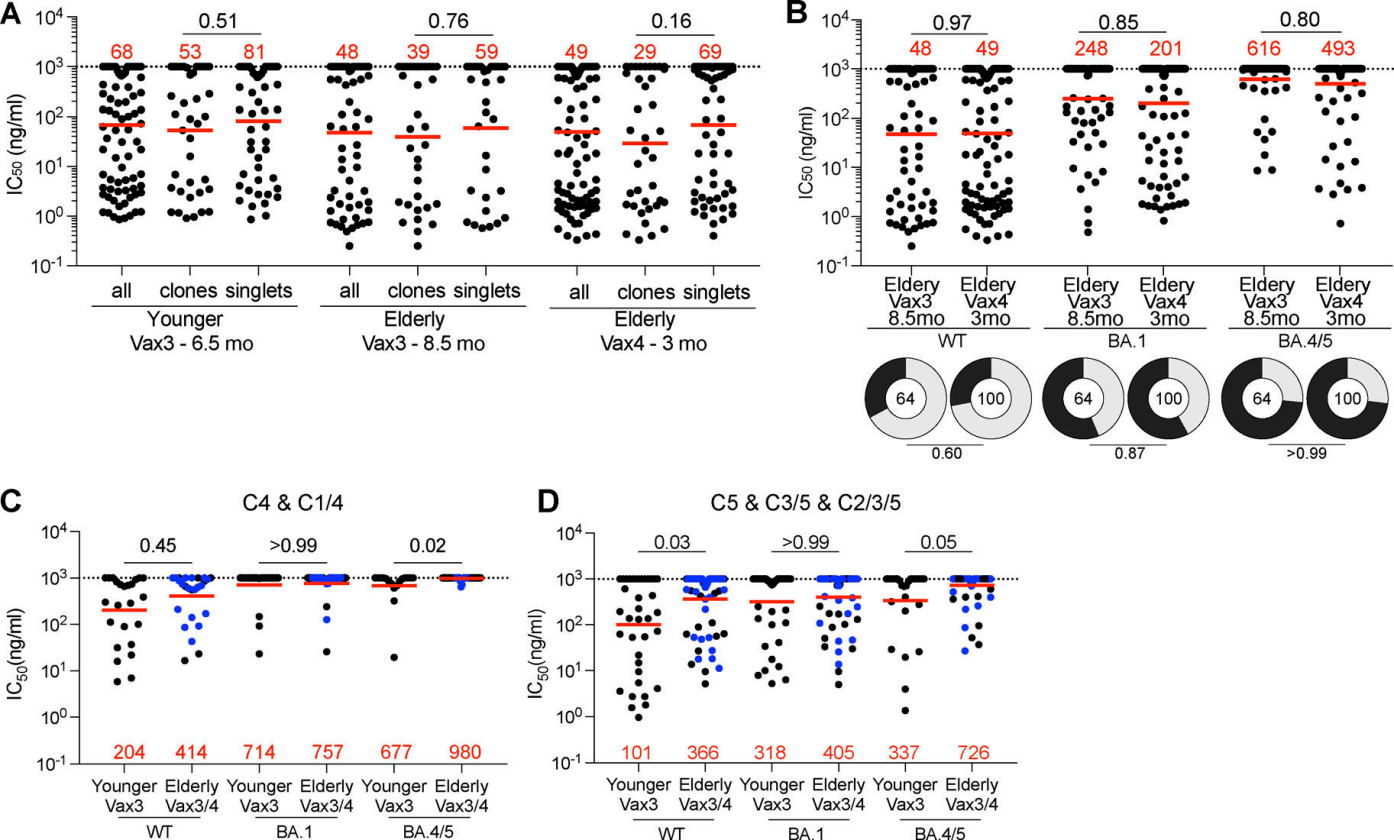
**mAb neutralizing breadth. (A)** Graphs show anti-SARS-CoV-2 neutralizing activity (IC_50_s) of mAbs (*n* = 253) measured by a SARS-CoV-2 pseudotype virus neutralization assay using WT spike, for all tested antibodies, clones, and singlets. **(B)** Graphs show anti-SARS-CoV-2 neutralizing activity (IC_50_s) of mAbs isolated from the elderly vaccinees after Vax3 or Vax4, against WT, Omicron BA.1, and Omicron BA.4/5 SARS-CoV-2 pseudoviruses. Pie charts illustrate the fraction of neutralizing (IC_50_ < 1,000 ng/ml) antibodies (gray slices) and non-neutralizing (IC_50_ > 1,000 ng/ml) antibodies (dark slices), inner circle shows the number of antibodies tested per group. **(C and D)** Graphs showing IC_50_ neutralization activity of Class 4, 1/4 (C), and Class 5, 3/5, and 2/3/5 antibodies among all antibodies in Fig. 4 A. The deletions/substitutions corresponding to viral variants were incorporated into a spike protein that also includes the R683G substitution, which disrupts the furin cleavage site and increases particle infectivity. Neutralizing activity against mutant pseudoviruses was compared to a WT SARS-CoV-2 spike sequence (NC_045512), carrying R683G. All experiments were performed at least in duplicate and repeated twice. Each dot represents one antibody. Antibodies sequences from elderly Vax4 are shown in blue. Red bars and values represent geometric mean values. Horizontal bars and red numbers indicate geometric mean values. Statistical significance was determined by Mann–Whitney test and for the ring plots in B by two-sided Fisher’s exact test.

253 RBD-binding antibodies were tested for neutralizing activity in a SARS-CoV-2 pseudotype neutralization assay using WT, Omicron BA.1, and BA.4/5 SARS-CoV-2 spikes (Wang et al., 2022b). Although there was no significant difference in antibody potency against WT or variant pseudoviruses between elderly and younger individuals (Fig. 4, A and B; Fig. S5, A and B; and Table S3), the epitopes targeted by the neutralizing antibodies were different in the two groups. Neutralizing antibodies obtained from the elderly were slightly biased to recognize Class 1 and 2 epitopes irrespective of their breadth of activity against different variants (Fig. 4, C and D). In contrast, neutralizing antibodies that recognize Class 4 and 5 epitopes were enriched in younger individuals (Fig. 4 C and Fig. S5, C and D).

## Discussion

Memory B cells are essential contributors to rapid antibody production upon pathogen challenge (Kurosaki et al., 2015; Victora and Nussenzweig, 2022; Weisel and Shlomchik, 2017). Several clinical trials of passive antibody therapy have demonstrated that early administration of significant quantities of neutralizing antibodies is essential for averting the serious consequences of SARS-CoV-2 infection in susceptible individuals (Cohen, 2022; Focosi et al., 2022; Gupta et al., 2021; Hammond et al., 2022; Montgomery et al., 2022; Weinreich et al., 2021). Our data indicate that elderly individuals that received a third or fourth dose of mRNA vaccine developed smaller absolute numbers of SARS-CoV-2 RBD-specific memory B cells that express a more limited antibody repertoire than younger vaccinees. This limitation along with a more limited T cell repertoire in elderly individuals (Britanova et al., 2014; Goronzy and Weyand, 2019; Sun et al., 2022) could contribute to a blunted neutralizing antibody response to infection and increased risk of serious outcomes in the elderly (Cerqueira-Silva et al., 2022).

Aging is associated with several different defects that impact humoral immunity. These include decreased B cell production in the bone marrow (Labrie et al., 2004; Miller and Allman, 2003; Stephan et al., 1998; Zharhary, 1988), smaller numbers of circulating memory B cells (Frasca et al., 2011; Paganelli et al., 1992), more limited germinal center responses (Kosco et al., 1989; Luscieti et al., 1980; Sage et al., 2015; Shankwitz et al., 2020; Szakal et al., 1990), and alterations in signaling in both B and T cells (Frasca et al., 2020; Mogilenko et al., 2022), as well as reduced number of innate immune cells (Sohrabi et al., 2021). Each of these could contribute to our observation that the elderly develop fewer anti-SARS-CoV-2 RBD-specific memory B cells.

Despite the overall decrease in the number of memory B cells, the neutralizing activity of individual memory antibodies in the elderly was not significantly different from that of the younger cohort. However, the memory repertoire in the elderly continued to be dominated by Class 1/2 antibodies after three vaccine doses while younger individuals evolved to produce memory that was focused on other epitope classes, which include more conserved regions of the RBD that remain less mutated in current circulating variants (Cao et al., 2022; Muecksch et al., 2022). This observation is consistent with the finding that the immune response to influenza is also less adaptable in elderly individuals (Henry et al., 2019). Why the focus of the anti-RBD response fails to diversify in older individuals has not been determined but could be a combination of the more limited B cell numbers, altered signaling, and germinal center responses. In addition, diversification by epitope masking may be impaired due to lower levels of serum antibodies produced after the first and second vaccine doses (Collier et al., 2021; Schaefer-Babajew et al., 2023; Tas et al., 2022; Walsh et al., 2020).

Vaccination for influenza is specifically tailored to the elderly by increasing the dose (Grohskopf et al., 2022). Although an increased dose or a shorter interval between vaccinations for the elderly is not a currently recommended practice, our data suggest that modifying SARS-CoV-2 vaccine regimens specifically for this at-risk population should be considered, especially if they enhance memory B and T cell responses.

## Materials and methods

### Study participants

Participants were healthy adults that had been vaccinated with three or four doses of an mRNA vaccine (mRNA-1273 [Moderna] or BNT162b2 [Pfizer]). The participants were categorized into two study groups, as age was of interest: elderly (75–91 yr old) and younger (23–66 yr old). The elderly participants were followed up for a blood sample 8.5 or 3 mo after receiving their third or fourth dose of the mRNA vaccine, separately. The younger participants were followed up at 6.5 mo for a blood sample after the third dose. All participants provided written informed consent before participation in the study, and the study was conducted in accordance with Good Clinical Practice. The study was performed in compliance with all relevant ethical regulations, and the protocol (DRO-1006) for studies with human participants was approved by the Institutional Review Board of The Rockefeller University. For detailed participant characteristics, see Table S1.

### Blood samples processing and storage

Venous blood samples were collected in heparin and serum-gel monovette tubes by standard phlebotomy at The Rockefeller University. Peripheral blood mononuclear cells (PBMCs) obtained from samples collected were further purified as previously reported by gradient centrifugation and stored in liquid nitrogen in the presence of FCS and DMSO (Gaebler et al., 2021; Robbiani et al., 2020). Heparinized serum and plasma samples were aliquoted and stored at −20°C or less. Prior to experiments, aliquots of plasma samples were heat-inactivated (56°C for 1 h) and then stored at 4°C.

### ELISAs

ELISAs (Amanat et al., 2020) were performed to evaluate antibodies binding to SARS-CoV-2 WT (Wuhan-Hu-1) RBD, Omicron (BA.4/5) RBD, Omicron (XBB) RBD, and Omicron (XBB.1.5) RBD protein by a coating of high-binding 96-half-well plates (Corning 3690) with 50 μl per well of a 1 μg/ml indicated protein solution in PBS for 1 h at room temperature. Plates were washed six times with washing buffer (1× PBS with 0.05% Tween-20 [Sigma-Aldrich]) and incubated with 170 μl per well of blocking buffer (1× PBS with 1% BSA and 0.05% Tween-20 [Sigma-Aldrich] and 0.1 mM EDTA) for 1 h at room temperature. Immediately after blocking, plasma samples or mAbs were added to PBS and incubated for 1 h at room temperature. Plasma samples were assayed at a 1:66 starting dilution and 10 additional threefold serial dilutions. 10 μg/ml starting concentration was used to test mAbs followed by 10 additional fourfold serial dilutions. Plates were washed six times with washing buffer and then incubated with anti-human IgG secondary antibody conjugated to HRP (109-036-088 109-035-129; Jackson Immuno Research and A0295; Sigma-Aldrich) in blocking buffer at a 1:5,000 dilution. Plates were developed by the addition of the HRP substrate, 3,3′,5,5′-tetramethylbenzidine (Thermo Fisher Scientific) for 10 min (plasma samples and mAbs). 50 μl of 1 M H_2_SO_4_ was used to stop the reaction and absorbance was measured at 450 nm with an ELISA microplate reader (FluoStar Omega, BMG Labtech) with Omega and Omega MARS software for analysis. A positive control (for anti-RBD ELISA, plasma from participant COV72 [Robbiani et al., 2020], diluted 66.6-fold and 10 additional threefold serial dilutions in PBS; for anti-Omicron ELISA, plasma from B040 [Wang et al., 2022b] was used as a control) was added to every assay plate for normalization for plasma samples. The average of its signal was used for normalization of all the other values on the same plate with Excel software before calculating the half-maximal binding titer using four-parameter nonlinear regression (GraphPad Prism v.9.1). Negative controls of pre-pandemic plasma samples from healthy donors were used for validation (for more details, please see Robbiani et al., 2020). For mAbs, the EC_50_ was determined using four-parameter nonlinear regression (GraphPad Prism v.9.1). EC_50_s above 1,000 ng/ml were considered non-binders.

### SARS-CoV-2 pseudotyped reporter virus

The plasmid pSARS-CoV-2-S_Δ19_ expressing a C-terminally truncated SARS-CoV-2 spike protein based on Wuhan-Hu-1 spike has been described before (Robbiani et al., 2020; Schmidt et al., 2020). Variant pseudoviruses resembling SARS-CoV-2 variants Omicron BA.1, Omicron BA.2, Omicron BA.4/5, Omicron BA.2.75.2, and Omicron XBB.1.5 have been described before (Schmidt et al., 2022; Wang et al., 2022a, 2022b) and were generated by the introduction of substitutions using synthetic gene fragments (IDT) or overlap extension PCR-mediated mutagenesis and Gibson assembly. Specifically, the variant-specific deletions and substitutions introduced were as follows:

Omicron BA.1: A67V, Δ69-70, T95I, G142D, Δ143-145, Δ211, L212I, ins214EPE, G339D, S371L, S373P, S375F, K417N, N440K, G446S, S477N, T478K, E484A, Q493K, G496S, Q498R, N501Y, Y505H, T547K, D614G, H655Y, H679K, P681H, N764K, D796Y, N856K, Q954H, N969H, N969K, L981F.

Omicron BA.2: T19I, L24S, del25-27, G142D, V213G, G339D, S371F, S373P, S375F, T376A, D405N, R408S, K417N, N440K, S477N, T478K, E484A, Q493R, Q498R, N501Y, Y505H, D614G, H655Y, N679K, P681H, N764K, D796Y, Q954H, N969K, Omicron BA.4/5: T19I, L24S, del25-27, del69-70, G142D, V213G, G339D, S371F, S373P, S375F, T376A, D405N, R408S, K417N, N440K, L452R, S477N, T478K, E484A, F486V, Q498R, N501Y, Y505H, D614G, H655Y, N679K, P681H, N764K, D796Y, Q954H, N969K.

Omicron BA.2.75.2: T19I, L24S, del25-27, G142D, K147E, W152R, F157L, I210V, V213G, G257S, G339H, R346T, S371F, S373P, S375F, T376A, D405N, R408S, K417N, N440K, G446S, N460K, S477N, T478K, E484A, F486S, Q498R, N501Y, Y505H, D614G, H655Y, N679K, P681H, N764K, D796Y, Q954H, N969K, D1199N; Omicron XBB.1.5: T19I, L24S, del25-27, V83A, G142D, del144, H146Q, Q183E, V213E, G252V, G339H, R346T, L368I, S371F, S373P, S375F, T376A, D405N, R408S, K417N, N440K, V445P, G446S, N460K, S477N, T478K, E484A, F486P, F490S, Q498R, N501Y, Y505H, D614G, H655Y, N679K, P681H, N764K, D796Y, Q954H, N969K.

Deletions/substitutions corresponding to variants of concern listed above were incorporated into a spike protein that also includes the R683G substitution, which disrupts the furin cleavage site and increases particle infectivity. Neutralizing activity against mutant pseudoviruses was compared to a WT SARS-CoV-2 spike sequence (NC_045512) carrying R683G.

SARS-CoV-2 pseudotyped particles were generated as previously described (Robbiani et al., 2020; Schmidt et al., 2020). Briefly, 293T (CRL-11268) cells were obtained from ATCC and transfected with pNL4-3ΔEnv-nanoluc and pSARS-CoV-2-S_Δ19_. The particles were harvested 48 h after transfection, filtered, and stored at −80°C.

### Pseudotyped virus neutralization assay

Prepandemic negative control plasma from healthy donors, plasma from individuals who received a third or fourth dose of an mRNA vaccine, or mAbs were fivefold serially diluted and incubated with SARS-CoV-2 pseudotyped virus for 1 h at 37°C. The mixture was subsequently incubated with HT1080/Ace2 cl14 cells for 48 h after which cells were washed with PBS and lysed with Luciferase Cell Culture Lysis 5× reagent (Promega). Nanoluc Luciferase activity in lysates was measured using the Nano-Glo Luciferase Assay System (Promega) with the ClarioStar Microplate Multimode Reader (BMG). The relative luminescence units were normalized to those derived from cells infected with SARS-CoV-2 pseudotyped virus (Wang et al., 2021c) in the absence of plasma or mAbs. The half-maximal neutralization titers for plasma (NT_50_) or half-maximal inhibitory concentrations for mAbs (IC_50_) were determined using four-parameter nonlinear regression (least squares regression method without weighting; constraints: top = 1, bottom = 0; GraphPad Prism).

### Biotinylation of viral protein for use in flow cytometry

Purified and Avi-tagged SARS-CoV-2 WT RBD was biotinylated using the Biotin-Protein Ligase-BIRA kit according to the manufacturer’s instructions (Avidity) as described before (Robbiani et al., 2020). Ovalbumin (A5503-1G; Sigma-Aldrich) was biotinylated using the EZ-Link Sulfo-NHS-LC-Biotinylation kit according to the manufacturer’s instructions (Thermo Fisher Scientific). Biotinylated ovalbumin was conjugated to streptavidin-BV711 for single-cell sorts (563262; BD biosciences). WT RBD was conjugated to streptavidin-PE (554061; BD Biosciences) and streptavidin-AF647 (405237; BioLegend) for single-cell sorting.

### Flow cytometry and single-cell sorting

Single-cell sorting by flow cytometry was described previously (Robbiani et al., 2020). Simply, PBMCs were enriched for B cells by negative selection using a pan–B cell isolation kit according to the manufacturer’s instructions (130-101-638; Miltenyi Biotec). The enriched B cells were incubated in FACS buffer (1× PBS, 2% FCS, 1 mM EDTA) with the following anti-human antibodies (all at 1:200 dilution): anti-CD20-PECy7 (335793; BD Biosciences), anti-CD3-APC-eFluro 780 (47-0037-41; Invitrogen), anti-CD8-APC-eFluor 780 (47-0086-42; Invitrogen), anti-CD16-APC-eFluor 780 (47-0168-41; Invitrogen), anti-CD14-APC-eFluor 780 (47-0149-42; Invitrogen), as well as Zombie NIR (423105; BioLegend) and fluorophore-labeled RBD and ovalbumin (Ova) for 30 min on ice. Single CD3^−^CD8^−^CD14^−^CD16^−^CD20^+^Ova^−^WT RBD-PE^+^-WT RBD-AF647^+^ B cells were sorted into individual wells of 96-well plates containing 4 μl of lysis buffer (0.5 × PBS, 10 mM dithiothreitol, 3,000 units/ml RNasin Ribonuclease Inhibitors [N2615; Promega]) per well using a FACS Aria III and FACSDiva software (Becton Dickinson) for acquisition and FlowJo for analysis. The sorted cells were frozen on dry ice and then stored at −80°C or immediately used for subsequent RNA reverse transcription. For B cell phenotype analysis, in addition to above antibodies, B cells were also stained with the following anti-human antibodies (all at 1:200 dilution): anti-IgD-BV650 (740594; BD), anti-CD27-BV786 (563327; BD biosciences), anti-CD19-BV605 (302244; BioLegend), anti-CD71-PerCP-Cy5.5 (334114; BioLegend), anti-IgG-PECF594 (562538; BD), anti-IgM-AF700 (314538; BioLegend), anti-IgA-Viogreen (130-113-481; Miltenyi Biotec), anti-CD11c-BV711(563130; BD biosciences), anti-CD21^−^ PerCPCy5.5 (354908; BioLegend), and anti-CD38-BV421 (562445; BD biosciences).

### Antibody sequencing, cloning, and expression

Antibodies were identified and sequenced as described previously (Robbiani et al., 2020; Wang et al., 2021a). In brief, RNA from single cells was reverse transcribed (18080-044; SuperScript III Reverse Transcriptase, Invitrogen) and the cDNA was stored at −20°C or used for subsequent amplification of the variable *IGH*, *IGL*, and *IGK* genes by nested PCR and Sanger sequencing. Sequence analysis was performed using MacVector. Amplicons from the first PCR reaction were used as templates for sequence- and ligation-independent cloning into antibody expression vectors. Recombinant mAbs were produced and purified as previously described (Robbiani et al., 2020).

### 10x Genomics

All procedures were performed while maintaining cells at 4°C. B cells were negatively selected from PBMCs with a pan–B cell isolation kit. 10x Genomics V(D)J libraries were generated with the Chromium Single Cell 5′ Library & Gel Bead Kit (10x Genomics; cat. PN-1000014) and Chromium Single Cell V(D)J Enrichment Kit, Human B cell (10x Genomics; cat. PN-1000016) as described in the 10x Genomics protocol. The 5′ expression library was sequenced with NovaSeq 6000 S1 (100 cycles; cat. 20012865; Illumina) and the V(D)J library was sequenced with NextSeq 500/550 Mid Output Kit v2.5 (300 cycles; cat. 20024905; Illumina).

### Single-cell RNA sequencing processing

The UMI quantification and BCR clonotype assembly were performed using CellRanger (v.7.1.0) and analyzed in R with Seurat (v.4.3.0). Cells with a mitochondrial proportion >10% and/or a feature count <200 or >2,500 were discarded. Sample batches were combined, normalized, and scaled with SCTransform. Based on their gene expression profile, single cells were visualized in a lower dimensional space using Uniform Manifold Approximation and Projection (UMAP) clustering.

### Biolayer interferometry

Biolayer interferometry assays were performed as previously described (Robbiani et al., 2020). In brief, we used the Octet Red instrument (ForteBio) at 30°C with shaking at 1,000 rpm. Epitope binding assays were performed with protein A biosensor (18-5010; ForteBio), following the manufacturer’s protocol “classical sandwich assay” as follows: (1) Sensor check: sensors immersed 30 s in buffer alone (18-1105; buffer ForteBio); (2) capture first antibody: sensors immersed 10 min with Ab1 at 10 μg/ml; (3) baseline: sensors immersed 30 s in buffer alone; (4) blocking: sensors immersed 5 min with IgG isotype control at 10 μg/ml; (5) baseline: sensors immersed 30 s in buffer alone; (6) antigen association: sensors immersed 5 min with RBD at 10 μg/ml; (7) baseline: sensors immersed 30 s in buffer alone; (8) association Ab2: sensors immersed 5 min with Ab2 at 10 μg/ml. Curve fitting was performed using the Fortebio Octet Data analysis software (ForteBio). Affinity measurements of anti-SARS-CoV-2 IgGs binding were corrected by subtracting the signal obtained from traces performed with IgGs in the absence of WT RBD. The kinetic analysis using protein A biosensor (as above) was performed as follows: (1) baseline: 60 s immersion in buffer; (2) loading: 200 s immersion in a solution with IgGs 10 μg/ml; (3) baseline: 200 s immersion in buffer; (4) association: 300 s immersion in solution with WT RBD at 20, 10 or 5 μg/ml; (5) dissociation: 600 s immersion in buffer. Curve fitting was performed using a fast 1:1 binding model and the data analysis software (ForteBio). Mean *K*_D_ values were determined by averaging all binding curves that matched the theoretical fit with an R^2^ value ≥0.8.

### Computational analyses of antibody sequences

Antibody sequences were trimmed based on quality and annotated using Igblastn v.1.14 with IMGT domain delineation system. Annotation was performed systematically using Change-O toolkit v.0.4.540 (Gupta et al., 2015). The clonality of heavy and light chains was determined using DefineClones.py implemented by Change-O v.0.4.5 (Gupta et al., 2015). The script calculates the Hamming distance between each sequence in the data set and its nearest neighbor. Distances are subsequently normalized, and to account for differences in junction sequence length, clonality is determined based on a cut-off threshold of 0.15. Heavy and light chains derived from the same cell were subsequently paired and clonotypes were assigned based on their V and J genes using in-house R and Perl scripts. All scripts and the data used to process antibody sequences are publicly available on GitHub (https://github.com/stratust/igpipeline/tree/igpipeline2_timepoint_v2).

The frequency distributions of human V genes in anti-SARS-CoV-2 antibodies from this study were compared to 131,284,220 IgH and IgL sequences generated by Soto et al. (2019) and downloaded from cAb-Rep (Guo et al., 2019), a database of human shared BCR clonotypes available at https://cab-rep.c2b2.columbia.edu/. We selected the IgH and IgL sequences from the database that are partially coded by the same V genes and counted them according to the constant region. The frequencies shown in Fig. S4 are relative to the source and isotype analyzed. We used the two-sided binomial test to check whether the number of sequences belonging to a specific *IGHV* or *IGLV* gene in the repertoire is different according to the frequency of the same IgV gene in the database. Adjusted P values were calculated using the false discovery rate correction. Significant differences are denoted with stars.

Nucleotide somatic hypermutation and complementarity-determining region (CDR3) length were determined using in-house R and Perl scripts. For somatic hypermutations, *IGHV* and *IGLV* nucleotide sequences were aligned against their closest germlines using Igblastn, and the number of differences was considered to correspond to nucleotide mutations. The average number of mutations for V genes was calculated by dividing the sum of all nucleotide mutations across all participants by the number of sequences used for the analysis.

### Data presentation

Figures were arranged in Adobe Illustrator (2022).

### Online supplemental material

Fig. S1 shows the correlation between plasma anti-RBD binding activity and vaccine dosing interval, or age. Fig. S2 shows flow cytometry gating strategy to phenotype or sort RBD-binding memory B cells after booster vaccination in the elderly and younger individuals. Fig. S3 shows frequency of V gene usage of RBD-binding memory B cells after vaccination or peripheral B cells in the elderly and younger individuals. Fig. S4 shows additional characterization of antibodies’ binding activity, epitopes, and somatic hypermutations. Fig. S5 shows additional characterization of antibodies’ neutralizing breadth. Table S1 details the individual characteristics for mRNA-vaccinated participants. Table S2 details sequence information of all characterized RBD-binding memory B cells from mRNA-vaccinated individuals. Table S3 provides information of a selected number of recombinant mAbs cloned from RBD-binding B cells.

## Supplementary Material

Table S1 details of the individual characteristics of mRNA-vaccinated participants.

Table S2 details sequence information of all characterized RBD-binding memory B cells from mRNA-vaccinated individuals.

Table S3 provides information on a selected number of recombinant mAbs cloned from RBD-binding B cells.

## Data Availability

Data are provided in Tables S1, S2, and S3. The raw sequencing data and computer scripts associated with Fig. 2 have been deposited at Github (https://github.com/stratust/igpipeline/tree/igpipeline2_timepoint_v2), and single-cell sequencing data is deposited in GSE233230. This study also uses data from “A Public Database of Memory and Naive B-Cell Receptor Sequences” (https://doi.org/10.5061/dryad.35ks2), PDB (6VYB and 6NB6), cAb-Rep (https://cab-rep.c2b2.columbia.edu/), Sequence Read Archive (accession SRP010970), and from “High frequency of shared clonotypes in human B cell receptor repertoires” (Soto et al., 2019). Computer code to process the antibody sequences is available at GitHub (https://github.com/stratust/igpipeline/tree/igpipeline2_timepoint_v2).
